# Comprehensive pan-cancer analysis of expression profiles and prognostic significance for NUMB and NUMBL in human tumors

**DOI:** 10.1097/MD.0000000000034717

**Published:** 2023-09-01

**Authors:** Yue Zhang, Hongxia Yang, Weizhe Liu, Qiuhang Song, Yunfeng Li, Juanjuan Zhang, Dingyan Zhou, Aiying Li

**Affiliations:** a Department of Biochemistry and Molecular Biology, College of Basic Medicine, Hebei University of Chinese Medicine, Shijiazhuang, Hebei, China; b Hebei Higher Education Institute Applied Technology Research Center on TCM Formula Preparation, Shijiazhuang, Hebei, China; c Hebei Key Laboratory of Chinese Medicine Research on Cardio-Cerebrovascular Disease, Shijiazhuang, Hebei, China; d Department of Clinical Foundation of Chinese Medicine, College of Basic Medicine, Hebei University of Chinese Medicine, Shijiazhuang, China.

**Keywords:** gene mutation, NUMB, NUMBL, pan-cancer, phosphorylation, prognosis

## Abstract

NUMB has been initially identified as a critical cell fate determinant that modulates cell differentiation via asymmetrical partitioning during mitosis, including tumor cells. However, it remains absent that a systematic assessment of the mechanisms underlying NUMB and its homologous protein NUMBLIKE (NUMBL) involvement in cancer. This study aimed to investigate the prognostic significance for NUMB and NUMBL in pan-cancer. In this study, using the online databases TIMER2.0, gene expression profiling interactive analysis, cBioPortal, the University of ALabama at Birmingham CANcer data analysis Portal, SearchTool for the Retrieval of Interacting Genes/Proteins, and R software, we focused on the relevance between NUMB/NUMBL and oncogenesis, progression, mutation, phosphorylation, function and prognosis. This study demonstrated that abnormal expression of NUMB and NUMBL were found to be significantly associated with clinicopathologic stages and the prognosis of survival. Besides, genetic alternations of NUMB and NUMBL focused on uterine corpus endometrial carcinoma, and higher genetic mutations of NUMBL were correlated with more prolonged overall survival and disease-free survival in different cancers. Moreover, S438 locus of NUMB peptide fragment was frequently phosphorylated in 4 cancer types and relevant to its phosphorylation sites. Furthermore, endocytosis processing and neurogenesis regulation were involved in the functional mechanisms of NUMB and NUMBL separately. Additionally, the pathway enrichment suggested that NUMB was implicated in Hippo, Neurotrophin, Thyroid hormone, and FoxO pathways, while MAPK, Hippo, Rap1, mTOR, and Notch pathways were related to the functions of NUMBL. This study highlights the predictive roles of NUMB and NUMBL in pan-cancer, suggesting NUMB and NUMBL might be served as potential biomarkers for diagnosis and prognosis in various malignant tumors.

## 1. Introduction

Malignant cancers, on account of high incidence of relapse and poor prognosis, are one of the leading causes of death worldwide.^[1]^ Despite the consistent exertion and process executed in oncological research, such as surgery, radiotherapy, and chemotherapy, numerous tumors still remain incurable due to the lack of efficient therapeutic targets.^[2]^ Hence, it is imperative to identify robust prognostic biomarkers and provide optimal curative molecular targets for the devastating tumors. Owing to the tumor heterogeneity, it is necessary to conduct a pan-cancer expression analysis, which has been extensively used to shed more light on the common clinical features, heterogeneities, and prognostic survival of various human malignancies.^[3]^ Recently, pan-cancer analysis, presented by the cancer genome atlas (TCGA) and relevant online databases, provides the evaluation of different human cancer tissues at epigenomic, genomic, proteomic and transcriptomic levels.^[4]^ It is employed to distinguish specific functional genes and signaling pathways, contributing to a compositive and thorough understanding of human malignant tumors.^[5]^

NUMB and its mammalian homolog NUMBLIKE (NUMBL) belong to an evolutionarily conserved family of proteins, implicated in a variety range of important cellular processes, including cell fate determination, cell adhesion, cell migration, protein endocytosis and ubiquitination.^[6–11]^ In mammals neurogenesis, NUMBL primarily has redundant functions with NUMB.^[12]^ However, NUMB, identified initially as the cell fate determinant, shows asymmetric distribution in cells, yet, NUMBL exhibits to be symmetrically distributed in cytoplasm.^[13]^ In addition, NUMB is widely expressed during morphogenesis, whereas NUMBL is more restrictive to the central nervous system development.^[14]^ Accumulating evidence verifies that NUMB/NUMBL play critical roles in oncogenesis, tumor progression, and tumor metastasis.

To date, it remains controversial whether NUMB is characterized as a tumor suppressor gene or an oncogene. For one thing, downregulation of NUMB displays in several types of tumors, including breast, colorectal, oral, endometrial and lung cancer.^[15–19]^ The tumor suppressor function of NUMB is mainly involved in Notch signaling pathway inhibition, p53 stabilization, or Wnt signaling pathway stimulation, accompanied by the regulation mechanisms of epithelial–mesenchymal transition.^[17,20–22]^ Additionally, an increasing number of micro-RNAs also enhance oncogenicity by antagonizing NUMB, such as miR-31, miR-146, miR-543, miR-335, and miR-9.^[15,16,23,24]^ In contrast, experimental evidence indicates that NUMB has acted as an oncogenic modulator in hepatocellular carcinoma and lung adenocarcinoma and squamous cell carcinoma, by promoting cell proliferation, migration, and invasion, meanwhile.^[25,26]^ Therefore, it is still lacking that a systematic understanding of the biological roles and clinical significance of the NUMB protein in neoplasia of diversiform cancers.

Previous researches have established that the overexpression of NUMBL inhibits, while the elimination of NUMBL triggers the migration and invasion of glioma cells.^[27]^ Analogously, NUMBL deficiency, with no changes of NUMB expression, leads to increment in tumorigenic properties with a worse prognosis, based on NUMBL repressing the Notch pathway in breast, lung and colorectal tumors.^[28]^ On the contrary, another report has obtained that NUMBL also maintain multiple nodes of metastatic progression by persisting of cancer-initiating cells in advanced lung cancer.^[29]^ Consequently, studies undertaken so far provide conflicting evidence concerning the role of NUMBL in tumor progression. More importantly, it is of the utmost importance to investigate the similarity and divergence in function of NUMB and NUMBL regarding their pivotal roles in regulating tumorigenic progression and correlative molecular mechanisms.

Herein, a comprehensive pan-cancer analysis was systematically conducted, for the first time, that the relationship between NUMB/NUMBL and patient clinicopathological characteristics by using datasets, consisting of TIMER, Gene expression profiling interactive analysis (GEPIA), cBioPortal, the University of ALabama at Birmingham CANcer data analysis Portal (UALCAN), SearchTool for the Retrieval of Interacting Genes/Proteins (STRING), and R software. Then the links between NUMB/NUMBL and tumor pathogenesis were further explored, containing differential gene expression, pathological features, prognostic survival, genetic mutation, protein phosphorylation, and relative cellular pathway. Consequently, these results furnish novel expectations into the functional roles of NUMB/NUMBL in diversified human malignancies, emphasizing the potential molecular mechanisms of NUMB/NUMBL in the tumorigenicity and clinical prognosis in different cancers.

## 2. Materials and Methods

### 2.1. Ethics approval

The study has been approved by the Institutional Animal Care and Use Committee of Hebei University of Chinese Medicine (No. DWLL2020063). And the data retrieved from the online datasets could be confirmed that all written informed consent had already been obtained.

### 2.2. TIMER

TIMER: Tumor Immune Estimation Resource (https://cistrome.shinyapps.io/timer) provides user-friendly analytic modules for dynamic analysis and visualization of a wide-spectrum of factors for systematical analysis of immune infiltrates across diverse cancer types, including gene expression, clinical outcomes, somatic mutations, and somatic copy number alterations.^[30]^ NUMB and NUMBL are selected to input via “Gene_module” and generated boxplots to visualize the expression difference of NUMB/NUMBL between tumor and normal tissues for the different tumor subtypes of the TCGA project.

### 2.3. GEPIA

The GEPIA database (http://gepia.cancer-pku.cn) is a web-based analytical server for unleashing the RNA sequencing expression data of 9736 tumors and 8587 normal samples from the big genomic data in TCGA and genotype-tissue expression datasets, using a standard processing pipeline. In this study, customizable differential gene expression was performed to assess comparison between various cancers and normal samples, pathological stage analysis, survival prognosis and correlation analysis provided by GEPIA. The threshold was selected as the default value.^[31]^

### 2.4. cBioPortal

The cBioPortal for Cancer Genomics (http://cbioportal.org) is a comprehensive open-access resource, providing customized data storage for exploring, visualizing, and analyzing multidimensional cancer genomics data.^[32]^ Based on TCGA dataset, genetic mutations in *NUMB*/*NUMBL* and their associations with overall survival (OS) and disease-free survival (DFS) of patients were obtained from cBioPortal.

### 2.5. UALCAN

The UALCAN dataset (http://ualcan.path.uab.edu/) provides level 3 RNA-seq and clinical data from 31 cancer types from TCGA database, estimating the effect of potential gene expression level and clinicopathologic features.^[33]^ In this study, UALCAN was utilized to analyze phosphorylation of NUMB/NUMBL between normal tissue and primary tissues of selected tumors.

### 2.6. STRING

STRING (https://string-db.org/) is an online database of protein–protein interaction (PPI) information analysis, achieving a comprehensive and objective global network presented with a unique set of computational predictions. A PPI network analysis was applied to collect and integrate associations of NUMB/NUMBL and their potential co-expressed genes through STRING.

### 2.7. R software

Gene ontology (GO) and Kyoto encyclopedia of genes and genomes (KEGG) enriched pathways were finally performed with the “ggplot2” and “Cluster Profiler” R packages to extract the canonical bioprocesses. The data for biological process (BP), cellular component (CC), and molecular function (MF) were visualized as dotplots by using the dotplot function. The R language software Rstudio (Version 4.2.0) was utilized in this analysis. Two-tailed *P* < .05 indicated statistically significant enrichment. And 10 most enriched GO and 20 most enriched KEGG pathways were extracted.

## 3. Results

### 3.1. Aberrant transcriptional expression of NUMB/NUMBL in different cancers

**Figure 1.**
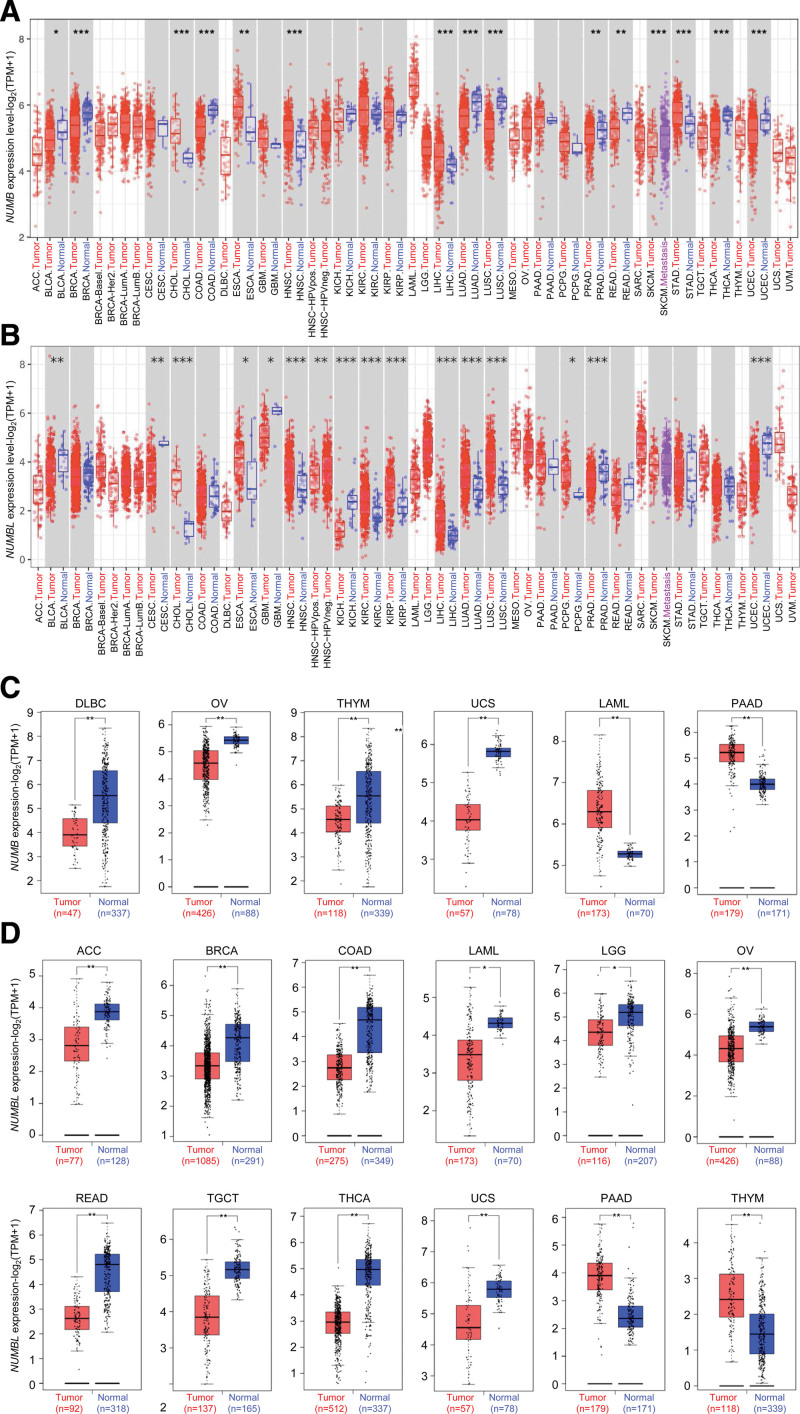
The relative expression level of *NUMB* and *NUMBL* in different cancers. (A and B) The expression status of the *NUMB* and *NUMBL* in pan-cancer, analyzed by TIMER2. (C and D) The mRNA expression of the *NUMB* and *NUMBL* in different cancer types between the tumor tissues and normal tissues, based on the cancer genome atlas (TCGA) and genotype-tissue expression (GTEx) data, analyzing by GEPIA2. Log_2_ (TPM + 1) was applied for log-scale. *P < .05, **P < .01, ***P < .001. GEPIA = gene expression profiling interactive analysis, NUMBL = NUMBLIKE.

To elucidate the significance of *NUMB*/*NUMBL* expressions in multiple tumor and normal tissue types of TCGA, mRNA expressions were analyzed by TIMER2 approach. As shown in Figure 1A, the relative mRNA expression level of *NUMB* was significantly declined relative to the corresponding normal controls in bladder urothelial carcinoma (BLCA), breast invasive carcinoma (BRCA), colon adenocarcinoma (COAD), lung adenocarcinoma (LUAD), lung squamous cell carcinoma (LUSC), prostate adenocarcinoma (PRAD), rectum adenocarcinoma (READ), skin cutaneous melanoma, thyroid carcinoma (THCA), and uterine corpus endometrial carcinoma (UCEC). In contrast, the expression of *NUMB* was observably elevated in cholangiocarcinoma (CHOL), esophageal carcinoma (ESCA), head and neck squamous cell carcinoma (HNSC), liver hepatocellular carcinoma (LIHC), and stomach adenocarcinoma (STAD), compared to normal controls.

After regarding the normal tissue of the genotype-tissue expression dataset as controls, relative expression level of *NUMB* was explored by GEPIA. In lymphoid neoplasm diffuse large B-cell lymphoma (DLBC), ovarian serous cystadenocarcinoma (OV), thymoma (THYM), and uterine carcinosarcoma (UCS), the expression of *NUMB* was lower than counterpart controls. In comparison, the expression level was higher than normal controls in acute myeloid leukemia (LAML) and pancreatic adenocarcinoma (PAAD) (Fig. 1C).

Simultaneously, in TIMER2 and GEPIA databases, it demonstrated that the mRNA expressions level of *NUMBL* remarkably down-regulated in adrenocortical carcinoma (ACC), BLCA, BRCA, COAD, cervical squamous cell carcinoma and endocervical adenocarcinoma, glioblastoma, kidney chromophobe (KICH), LAML, brain low grade glioma (LGG), OV, PRAD, READ, testicular germ cell tumor (TGCT), THCA, UCS, and UCEC. Furthermore, significant up-regulation of *NUMBL* was also found in CHOL, ESCA, HNSC, kidney renal clear cell carcinoma (KIRC), kidney renal papillary cell carcinoma (KIRP), LIHC, LUAD, LUSC, THYM, PAAD, and pheochromocytoma and paraganglioma (Fig. 1B and D). It is differential that the expression status of *NUMB* and *NUMBL* across various cancer types of TCGA.

### 3.2. Relationship between the mRNA expression of NUMB/NUMBL and the clinicopathological parameters of patients in multiple tumor types

**Figure 2.**
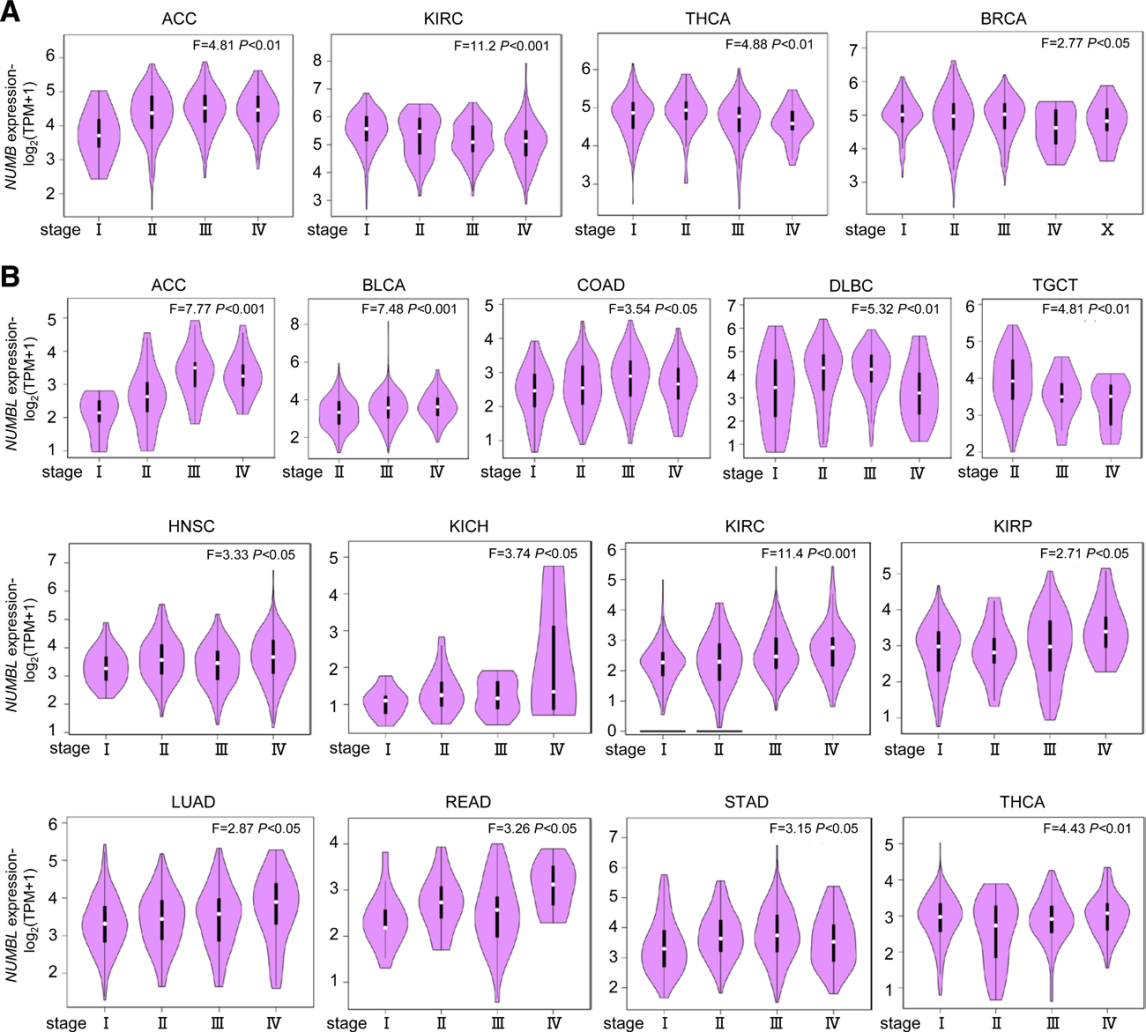
Correlation between *NUMB*/*NUMBL* expression and tumor pathological stages in different cancers. (A and B) The expression levels of the *NUMB*/*NUMBL* were in the tumor main pathological stages (stage I, stage II, stage III, and stage IV) of different cancers, analyzed by GEPIA. Log_2_ (TPM + 1) was applied for log-scale. GEPIA = gene expression profiling interactive analysis, NUMBL = NUMBLIKE.

Using the GEPIA, the correlations between the mRNA levels of *NUMB*/*NUMBL* with patients individual tumor stages of different cancers from TCGA were compared. The results indicated that the expression levels of *NUMB* markedly varied in ACC, BLCA, COAD, DLBC, and TGCT (Fig. 2A), whereas the expression levels of *NUMBL* appreciably diverged in ACC, BLCA, COAD, DLBC, HNBC, KICH, KIRC, KIRP, LUAD, READ, STAD, TGCT, and THCA (Fig. 2B).

In short, the observations above illustrated that relative mRNA expressions of *NUMB*/*NUMBL* were significantly related to patients individual cancer pathological stages. What is more, these data suggested that *NUMB*/*NUMBL* might play crucial roles in the tumor progression of different cancers from TCGA.

### 3.3. Prognostic features of the mRNA expression of NUMB/NUMBL with different cancers

**Figure 3.**
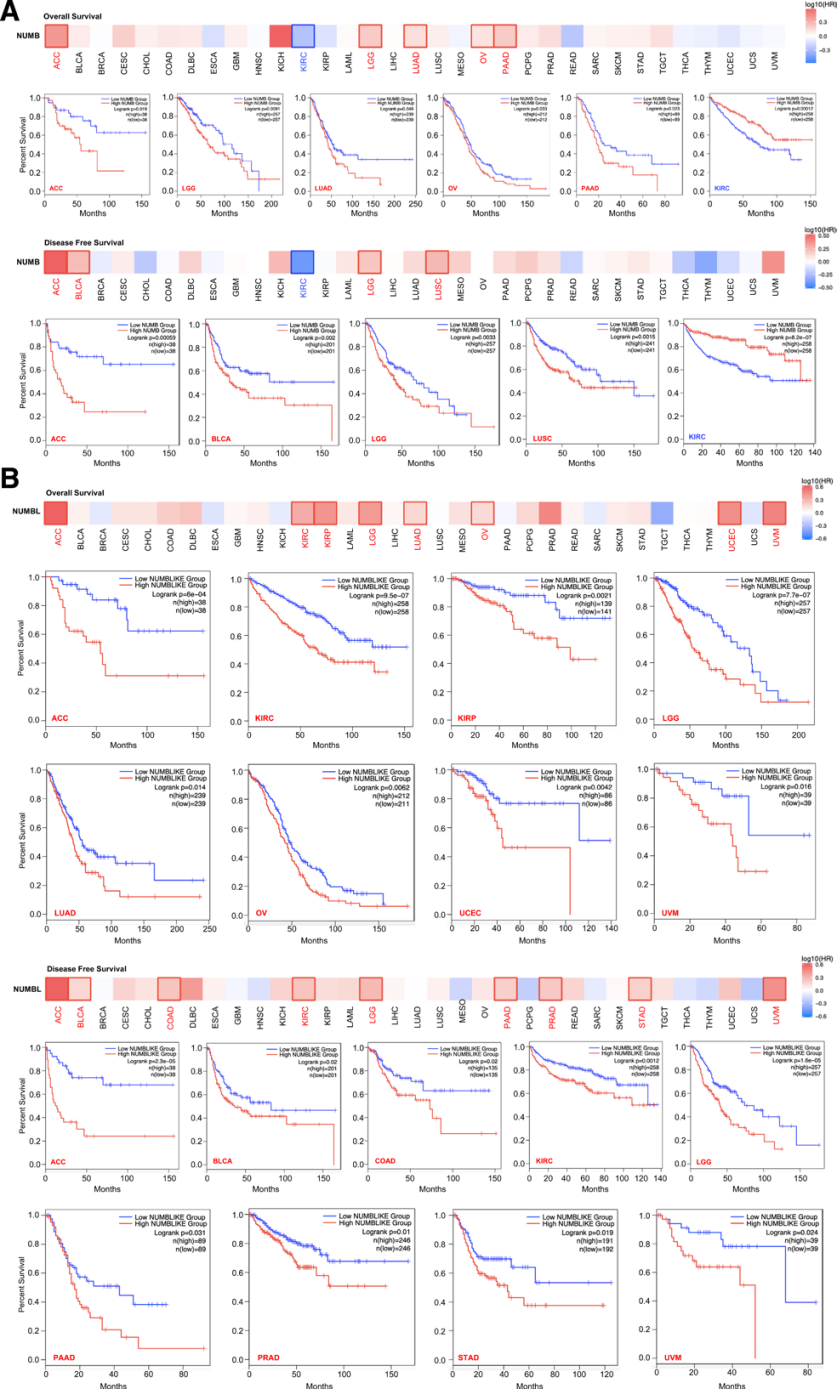
Correlation between *NUMB/NUMBL* expression and survival prognosis of different cancers in the cancer genome atlas (TCGA). The overall survival and disease free survival curves comparing patients with high (red) and low (blue) *NUMB* (A) or *NUMBL* (B) expression in different cancers were plotted using the GEPIA database at the threshold of *P* value of <.05. GEPIA = gene expression profiling interactive analysis, NUMBL = NUMBLIKE.

To evaluate the prognostic value of distinctively expressed *NUMB*/*NUMBL* with the progression of diverse cancers from TCGA, correlations between *NUMB*/*NUMBL* and clinical outcomes, including OS and DFS were investigated by GEPIA, respectively. In each cohort, patients were divided into low and high groups based on the expression cutoff value. It could be seen that, decreased *NUMB* mRNA expression was associated with poor prognosis of OS for cancers of ACC, LGG, LUAD, OV, and PAAD, and DFS for cancers of ACC, BLCA, LGG, and LUSC. By contrast, highly expressed *NUMB* led to an increased OS and DFS prognosis for KIRC (Fig. 3A).

In addition, Figure 3B revealed that low *NUMBL* mRNA expression was predicted to have poor OS prognosis for the TCGA cases of ACC, KIRC, KIRP, LGG, LUAD, OV, UCEC, and uveal melanoma, and DFS prognosis for ACC, BLCA, COAD, KIRC, LGG, PAAD, PRAD, STAD, and UVM.

### 3.4. Genetic mutations in NUMB/NUMBL and their associations with OS and DFS in diverse cancer types

**Figure 4.**
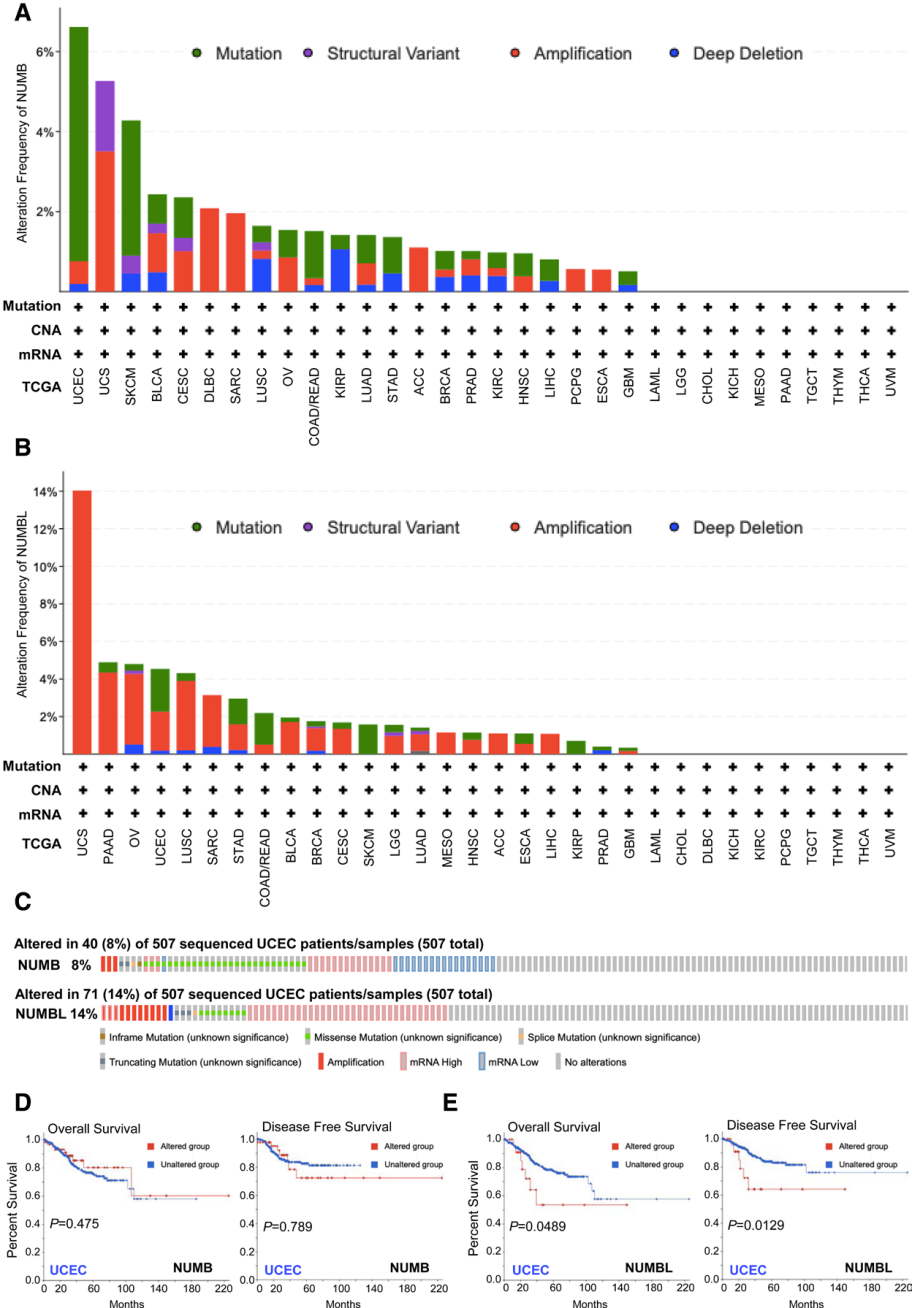
Genetic mutation features of *NUMB*/*NUMBL* in different cancers of the cancer genome atlas (TCGA). (A and B) The alteration frequency with mutation type of *NUMB*/*NUMBL*, using the cBioPortal tool. (C) Genetic alteration of *NUMB*/*NUMBL* in sequenced UCEC patients/samples. (D and E) The potential correlation between *NUMB*/*NUMBL* mutation status and overall or disease-free survival of UCEC, analyzed by cBioPortal. NUMBL = NUMBLIKE, UCEC = uterine corpus endometrial carcinoma.

Epigenetic alternations play an essential role in early tumor malignancies.^[34]^ Therefore, genetic mutations of NUMB/NUMBL in cancer patients were conducted by cBioPortal online tool. The results exhibited that the highest mutation frequency of NUMB (8%) and NUMBL (14%) appeared for patients with UCEC (Fig. 4A–C).

Furthermore, the relationship of genetic alternation in NUMB/NUMBL with OS and DFS of UCEC patients were analyzed by cBioPortal dataset. Results observed from the Kaplan–Meier plot and log-rank test revealed that the genetic mutation of NUMB had no significant relation to OS and DFS, whereas altered NUMBL correlated to lower OS and DFS of UCEC patients (Fig. 4D and E). These consequences discovered that the variation of NUMBL rather than NUMB might give rise to crucially affect the prognosis of UCEC patients.

### 3.5. Protein phosphorylation analysis of NUMB/NUMBL in certain tumors

In order to explore the association between protein phosphorylation and oncogenicity, the differences in NUMB/NUMBL phosphorylation levels between primary tumor tissues and normal tissues were compared. Utilizing the UALCAN database, 6 categories of tumors, including LUAD, OV, KIRC, BRCA, COAD, and UCEC, were analyzed.

**Figure 5.**
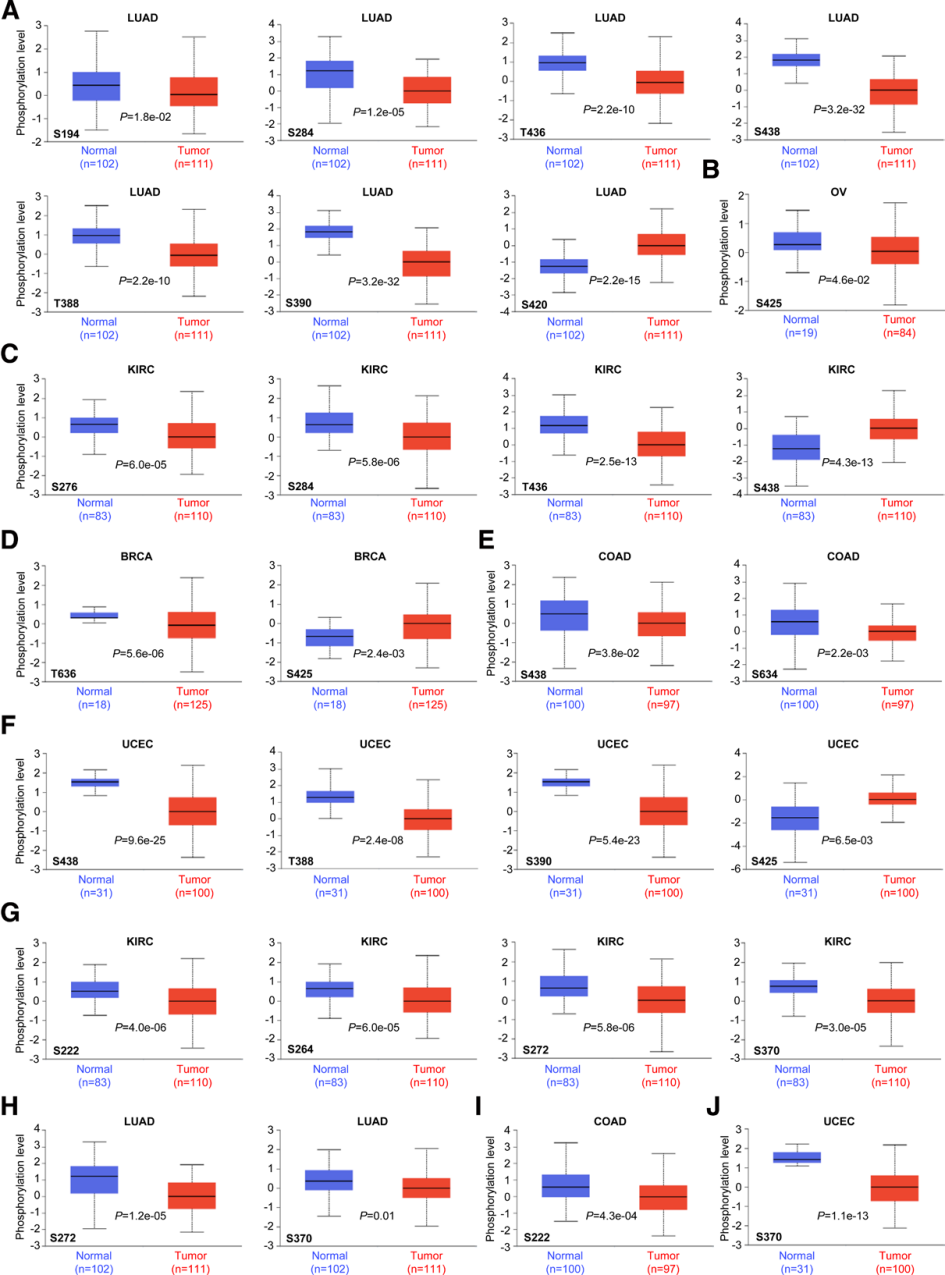
Phosphorylation analysis of NUMB/NUMBL in different tumors. The expression level of NUMB (A–F) phosphoprotein and NUMBL (G–J) phosphoprotein between normal tissues and primary tissue of selected tumors via the UALCAN. NUMBL = NUMBLIKE.

As shown in Figure 5A–F, phosphorylation of NUMB revealed significant differences between tumor and normal patients, especially S438 locus exhibited aberrant phosphorylation levels in 4 types of tumors (LUAD, KIRC, COAD, and UCEC) among all primary tumor tissues. Meanwhile, the remarkably differences of NUMBL phosphorylation levels between tumor and normal controls appeared in 4 types of primary tumors (KIRC, LUAD, COAD, and UCEC). Therein, S370 locus of NUMBL was frequently phosphorylated in KIRC, LUAD, and UCEC (Fig. 5G and H). These observations provided a further exploration of the potential roles of S438 phosphorylation of NUMB and S370 locus of NUMBL in tumor regulation.

### 3.6. Correlation analysis of NUMB- or NUMBL-related partner genes

**Figure 6.**
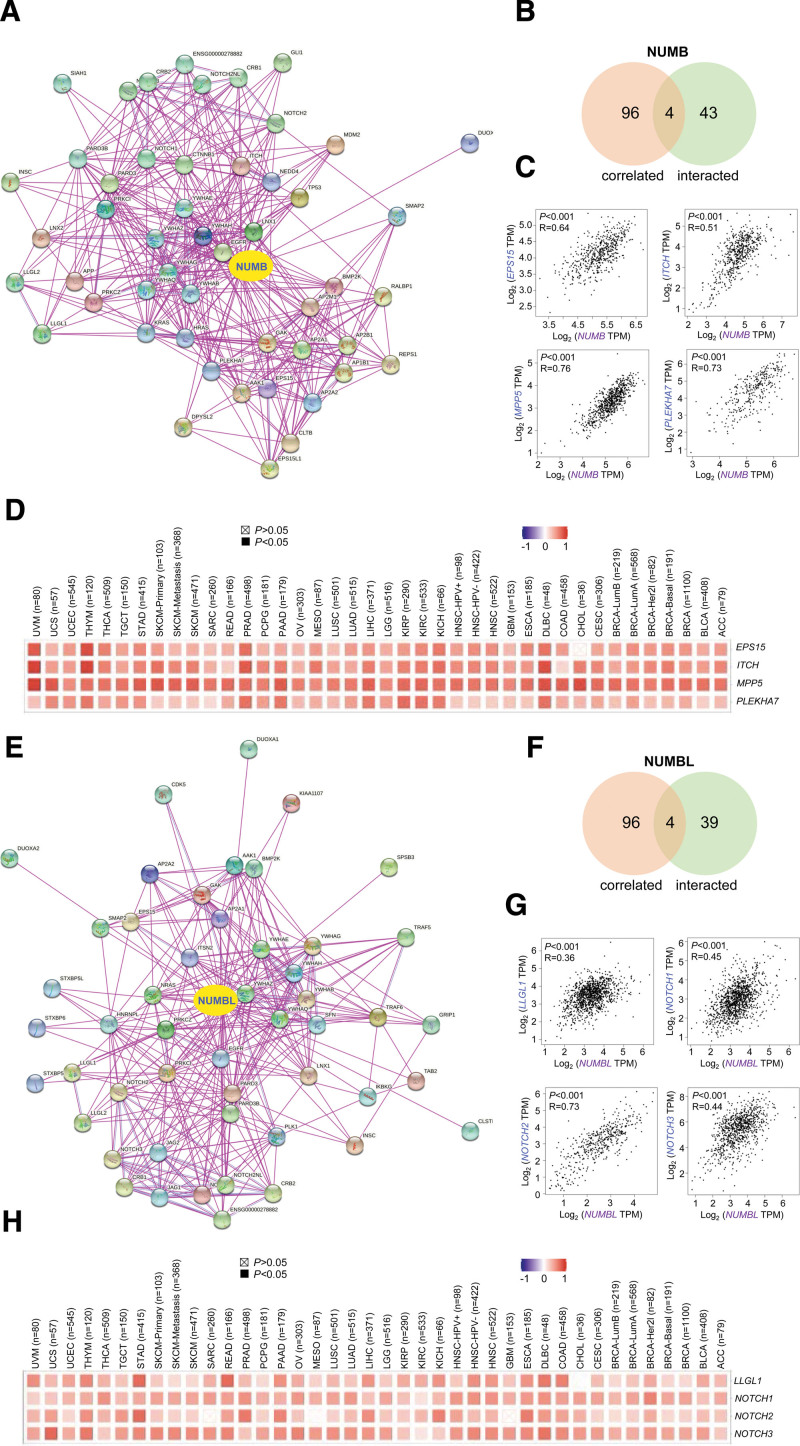
Analysis of NUMB- or NUMBL-related genes. (A and E) Network view of PPI for the related genes of NUMB/NUMBL (STRING). The nodes mean proteins, the edges mean the interaction of proteins. (B and F) The intersection analysis of the NUMB-/NUMBL-binding and correlated genes was conducted. (C and G) The top 100 NUMB-/NUMBL-correlated genes in the cancer genome atlas (TCGA) projects and analyzed the expression correlation between NUMB/NUMBL and selected targeting genes, analyzed by GEPIA. (D and H) The corresponding heatmap data in the detailed cancer types are displayed. GEPIA = gene expression profiling interactive analysis, NUMBL = NUMBLIKE.

To further investigate the molecular mechanisms of *NUMB*/*NUMBL* in oncogenesis and metastasis, NUMB- or NUMBL-related proteins were screened via STRING website. Based on the STRING instrument, the integrated network constructed by totally 50 NUMB- or NUMBL-interacted proteins supported by experimental evidence, separately (Fig. 6A and E). Besides, using the GEPIA dataset, top 100 genes in all tumor expression data of TCGA that correlated with *NUMB*- or *NUMBL* were gathered respectively.

An intersection analysis of the above 2 gene clusters indicated 4 overlapped members severally (Fig. 6B and F), containing *EPS15, ITCH, MPP5*, and *PLEKHA7* for *NUMB* and *LLGL1, NOTCH1, NOTCH2, NOTCH3*, and *NOTCH5* for *NUMBL*. Then the scatter diagrams showed that the *NUMB* expression level was positively correlated with *EPS15, ITCH, MPP5*, and *PLEKHA7*, while the *NUMBL* expression level was also positively correlated with *LLGL1, NOTCH1, NOTCH2*, and *NOTCH3* (Fig. 6C and G). Herein, the corresponding heatmap also showed a positive correlation between *NUMB* or *NUMBL* and their above targeting genes in the majority of detailed cancers (Fig. 6D and H).

### 3.7. Predicted functions and pathways of NUMB/NUMBL and their overlapping genes

After analyzing the correlated genes of NUMB/NUMBL in differing tumors from TCGA, the potential functions and pathways of NUMB/NUMBL and their associated genes were further predicted by GO and KEGG annotation. GO enrichment analysis represented the underlying functional roles of target host genes on the basis of 3 aspects, including BPs, CCs, and MFs.

**Figure 7.**
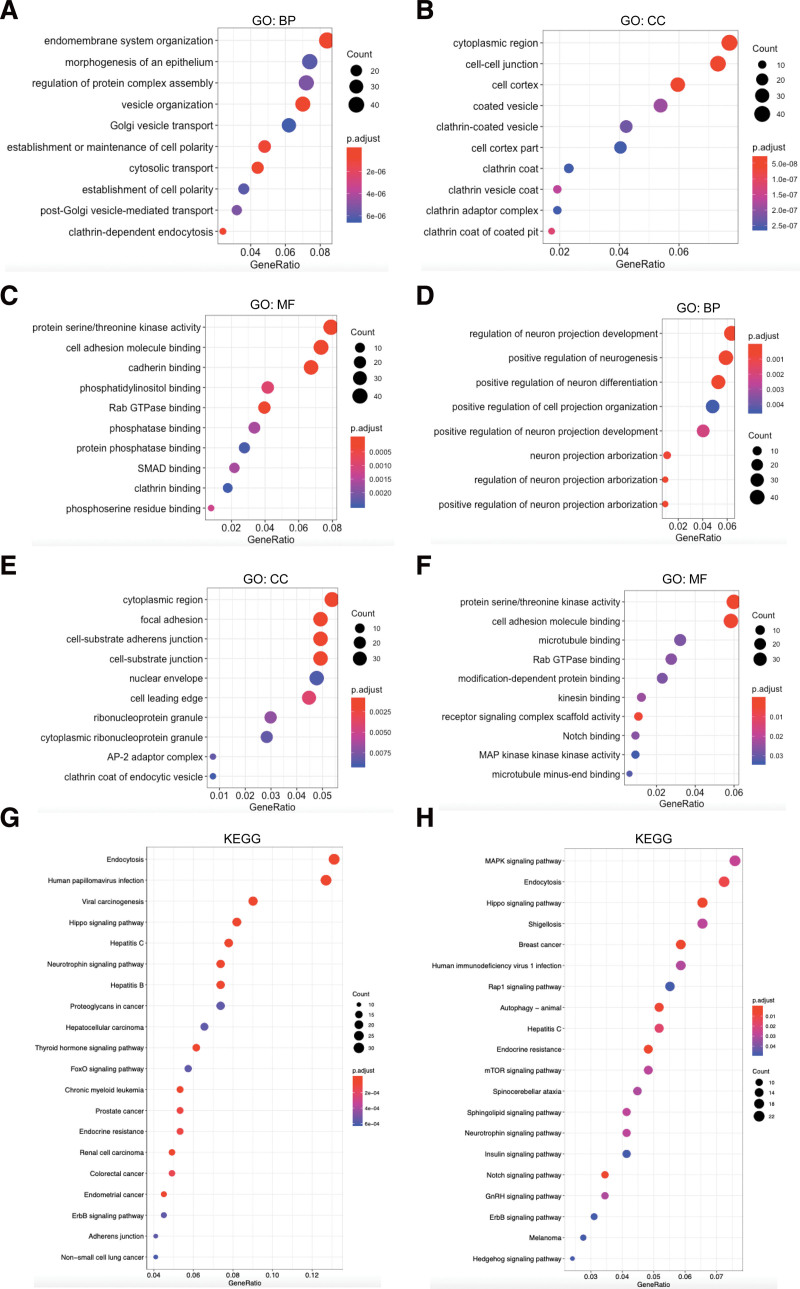
The functions of *NUMB*/*NUMBL* and genes significantly associated with *NUMB*/*NUMBL* alternations. (A–C and G) Gene ontology (GO) and Kyoto encyclopedia of genes and genomes (KEGG) enrichment analysis of *NUMB* and their co-expression genes. (D–F and H) GO and KEGG enrichment analysis of *NUMBL* and their co-expression genes. Biological processes (BP), cellular components (CC), molecular functions (MF) by Rstudio. NUMBL = NUMBLIKE.

As shown in Figure 7A, BPs associated with endomembrane system and endocytosis, such as endomembrane system organization, vesicle organization, Golgi vesicle transport, and post-Golgi vesicle-mediated transport were significantly regulated by NUMB alternations. While such BPs related to neurogenesis regulation, containing neuron projection development, regulation of neuron differentiation, and neuron projection arborization were also notably controlled by NUMBL alterations (Fig. 7D). Observably, CCs including cell-cell junction, cell cortex, and clathrin-coated vesicle were prominently affected by NUMB mutations (Fig. 7B). Meanwhile, focal adhesion, cell-substrate adherens junction, and nuclear envelope were greatly mediated by NUMBL mutations (Fig. 7E). Simultaneously, MFs including protein serine/threonine kinase activity, cell adhesion molecule binding, and Rab GTPase binding were remarkably related to both NUMB and NUMBL alterations (Fig. 7C and F).

**Figure 8.**
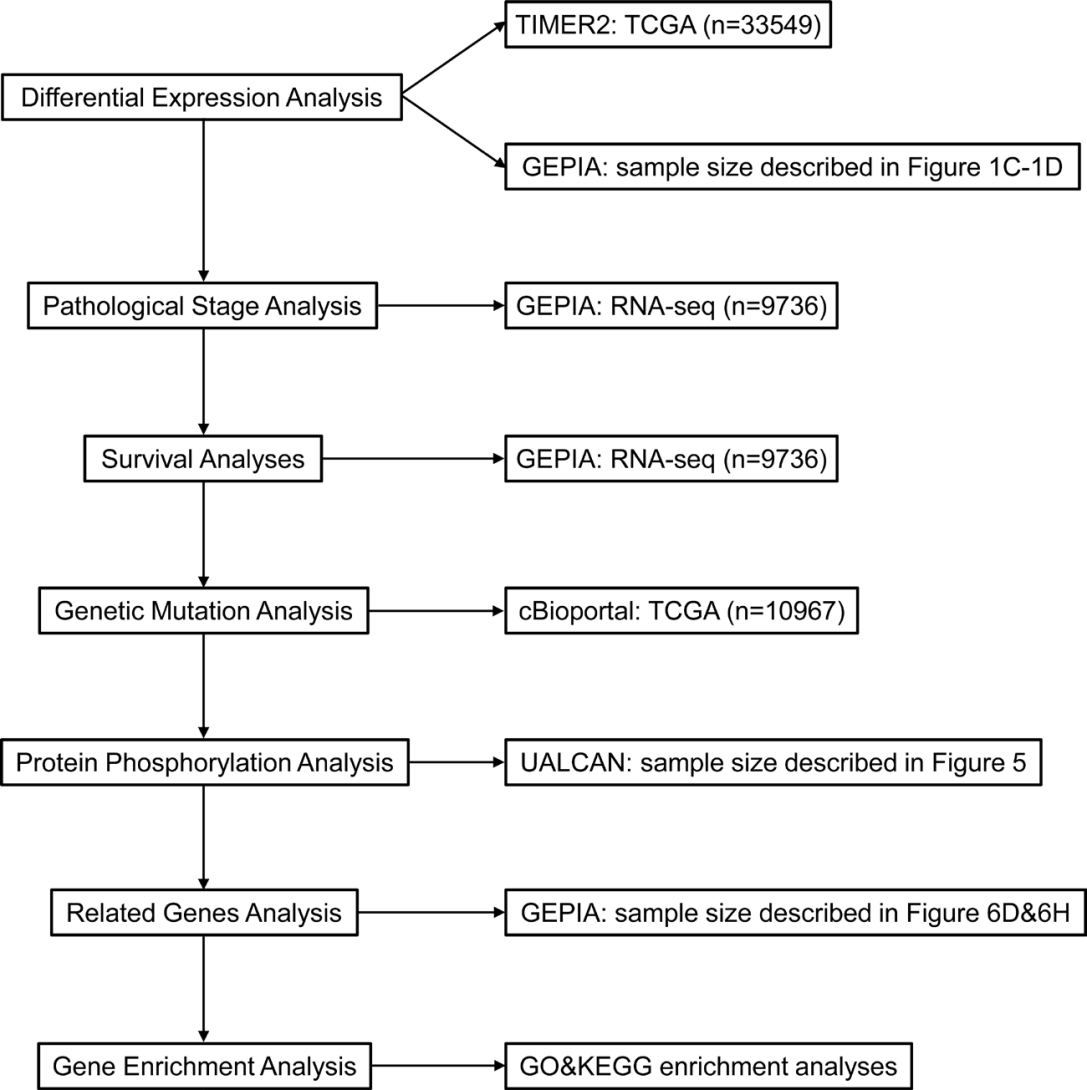
The flowchart of pan-cancer analysis. The workflow diagram for the methodology in this study as well as the steps and the number of cases including integration of datasets from various sources.

KEGG annotation can illuminate the pathways relevant to the functions of NUMB/NUMBL alterations and the frequently overlapping genes. In KEGG analysis, apart from multiple kinds of cancer, a number of signaling pathways, including Hippo, Neurotrophin, Thyroid hormone, and FoxO pathways, were associated with the functions of NUMB mutations (Fig. 7G). Moreover, MAPK, Hippo, Rap1, mTOR, Notch, ErbB, and Hedgehog signaling pathways were also correlated with the functions of NUMBL alterations (Fig. 7H). It was reported that those numerous signaling pathways above were involved in tumorigenesis and pathogenesis of malignant tumors. Finally, we utilized a flow chart to show the screening procedure of NUMB/NUMBL in pan-cancer more scrupulously (Fig. 8).

## 4. Discussion

Emerging publications have reported functional links between NUMB/NUMBL and clinicopathologic features in many types of cancers. Although the roles of NUMB/NUMBL in the tumorigenesis and prognosis of several cancers have been partially confirmed, further systematical analysis, especially pan-cancer analysis, of NUMB/NUMBL has yet to be elucidated. The present study, thus, is the first time to comprehensively examine the NUMB/NUMBL in a total of 33 categories of tumors based on the data of TCGA database and the molecular characteristics of relative gene expression, survival prognosis, genetic mutation, protein phosphorylation, and related signaling pathways.

Recent studies have revealed diminished expression of NUMB in majority tumors, and the absence of NUMB has been involved in promoting tumor growth, invasion, metastasis, and cell stemness.^[35–40]^ Firstly, NUMB has been regarded as a tumor suppressor by means of regulating several critical signaling pathways, including Notch, p53, Wnt, and Hedgehog.^[17,21,41,42]^ Moreover, prior studies have noted the essential role of NUMB in modulating epithelial–mesenchymal transition process, which is defined as inappropriately exploited during carcinogenesis, inducing the oncogenic transformation of cancer cells, making them prone to oncogenesis, migration, and invasion.^[43,44]^ What is more, being a characterized cell fate determinant during morphogenesis, NUMB also appears to control division mode in cancer stem cells, resulting in inhibition of their invasiveness and clonal and sphere formation capabilities.^[23]^ However, an increasing number of reports have uncovered that NUMB also acts as an oncogene in correlates with cell proliferation and poor prognosis in hepatocellular carcinoma patients.^[26]^ Interestingly, in lung cancer, how NUMB performs the function depends on the subtypes of tumors. As mentioned in the literature, high expression of NUMB was correlated with favorable prognosis in patients with lung adenocarcinoma rather than squamous cell carcinoma.^[25]^ Taken together, considering the complexity of NUMB, it should be dialectically elaborate the underlying mechanisms of NUMB functioning in tumorigenesis.

The evidence from this study implies that NUMB can have both tumor promoting and inhibiting capacities and those opposite impacts of NUMB might rely on tumor types. Through relative mRNA expression analysis via TIMER2 and GEPIA databases, our findings point that down-regulated expression of *NUMB* exhibits in 14 of 33 total cancers, consisting of BLCA, BRCA, COAD, DLBC, LUAD, LUSC, OV, PRAD, READ, skin cutaneous melanoma, THCA, THYM, UCEC, and UCS. Inversely, bioinformatic analysis also indicated significantly increased expression of *NUMB* in a few types of cancer, including CHOL, ESCA, HNSC, LAML, LIHC, PAAD, and STAD. Additionally, GEPIA data also reveals the considerable differences between pathological stages and *NUMB* mRNA expression in ACC, BLCA, COAD, DLBC, and TGCT, suggesting that *NUMB* may play several roles depending on distinct cell types and specific stages of development. Importantly, in most cancers, the upshot supports the idea that elevated *NUMB* expression predicts poorer patient prognosis. Only in KIRC, does lower *NUMB* expression correlate with poorer patient prognosis. Notably, an additional key finding in this study, by using cBioPortal dataset, is that the genetic alternation of NUMB performs an indispensable role in multiple cancer types, especially in UCEC. Further, we found frequent protein phosphorylation loci of NUMB in LUAD, OV, KIRC, BRCA, COAD, and UCEC, by utilizing UALCAN online tool, indicating the possible controlling sites of NUMB. Foremost, via STRING and GEPIA, co-expression genes of *NUMB* are collected to conduct *NUMB*-related partners, containing *EPS15, ITCH, MPP5*, and *PLEKHA7*. Based on collective overlapping genes, GO and KEGG enrichment clearly confirm that Hippo, Neurotrophin, Thyroid hormone, and FoxO pathways are closely interrelated to *NUMB* alternations. While, being distinguished from subsistent pathways, such as p53, Notch, Wnt, and Hedgehog, we have concluded novel signaling pathways underlying the molecular mechanisms of *NUMB* in tumor initiation, progression, and metastasis.

Compared to NUMB, less is known about the expression and role of NUMBL in most types of cancer until now. Unexpectedly, the analysis of expression level of *NUMBL* between tumor and normal tissues shows that dysregulation of *NUMBL* emerges in 27 of 33 tumor types of TCGA data library, more extensive than *NUMB*. Similarly, *NUMBL* also acts as both tumor suppressor and activator in various types of malignancies. Concerning the different stages of cancer patients, *NUMBL* relative expression exhibits remarkably variations in ACC, BLCA, COAD, DLBC, HNBC, KICH, KIRC, KIRP, LUAD, READ, STAD, TGCT, and THCA. Whereas the relevance between *NUMB* mRNA expression and pathological stages only presents in 5 tumor species. Dramatically, the increased *NUMBL* expression consistently predicts a poorer prognosis in both OS and DFS, which has imparities from *NUMB*. Furthermore, epigenetics mutation frequency of NUMBL is higher than NUMB among different cancers, and altered NUMBL, rather than NUMB, associated to lower OS and DFS of UCEC patients. Additionally, protein phosphorylation analysis demonstrates that the occurrence of NUMBL phosphorylation is less frequent than NUMB and the amount of phosphorylation loci of NUMBL is less than NUMB. Apparently, it has been investigated that overlapping genes of *NUMBL*, consisting of *LLGL1, NOTCH1, NOTCH2*, and *NOTCH3*, indicate the intimate interrelation between NUMBL and Notch signaling pathway. Notably, KEGG enrichment observation reveals the underlying mechanisms of NUMBL are implicated in MAPK, Hippo, Rap1, mTOR, Notch, ErbB, and Hedgehog pathways.

In accordance with our results, a pan-cancer analysis systematically investigates the role and function and relevant molecular mechanisms of NUMB and NUMBL in majority types of cancer, and discusses the similarities and divergences between these 2 homologous proteins. The pivotal finding is that the discrepancies of effects between NUMB and NUMBL on the signaling pathways. A possible explanation for this might be correlated to the alternative splicing of NUMB in tumors. It has been reported that NUMB has 4 splicing isoforms, while public databases may not take the alternative splicing into account, although they are robust tools to gain available data.

In summary, the results above anticipate that both NUMB and NUMBL may have values as promising biomarkers in detective and therapeutic measures of human malignancies. To date, the ultimate mechanisms of NUMB and NUMBL mediating cancer processes concentrate on breast,^[26,36]^ lung,^[25]^ colon,^[19]^ prostate,^[17,18]^ liver,^[24]^ and pancreatic^[23]^ cancers. Although we tried our best to make this research results accurate and reliable, the present study still has several limitations. Firstly, the sample size of some rare cancer types was limited, which may have led to erroneous results or ambiguous conclusions. Secondly, the current research mainly focused on bioinformatics analysis, and the prediction results need to be verified by further in vivo and in vitro experiments, such as real-time fluorescence quantitative polymerase chain reaction, western blot, and immunocytochemistry should be performed to understand specific functions of NUMB and NUMBL in carcinogenesis.

## Acknowledgements

We thank all authors who contributed valuable methods and data and made them public.

## Author contributions

**Conceptualization:** Yue Zhang, Hongxia Yang, Aiying Li.

**Data curation:** Yue Zhang.

**Formal analysis:** Weizhe Liu.

**Investigation:** Yunfeng Li.

**Methodology:** Qiuhang Song, Dingyan Zhou.

**Project administration:** Juanjuan Zhang.

**Resources:** Hongxia Yang, Aiying Li.

**Software:** Yue Zhang.

**Writing – original draft:** Yue Zhang.
